# Crystal structure and Hirshfeld surface analysis of (2*Z*)-3-oxo-*N*-phenyl-2-[(1*H*-pyrrol-2-yl)methylidene]butanamide monohydrate

**DOI:** 10.1107/S2056989023009799

**Published:** 2023-11-14

**Authors:** Ayten S. Safarova, Ali N. Khalilov, Mehmet Akkurt, Ivan Brito, Ajaya Bhattarai, Farid N. Naghiyev, Ibrahim G. Mamedov

**Affiliations:** a Department of Chemistry, Baku State University, Z. Khalilov str. 23, AZ1148 Baku, Azerbaijan; b "Composite Materials’ Scientific Research Center, Azerbaijan State Economic University (UNEC), H. Aliyev str. 135, AZ1063, Baku, Azerbaijan; c Department of Physics, Faculty of Sciences, Erciyes University, 38039 Kayseri, Türkiye; d Departamento de Química, Facultad de Ciencias Básicas, Universidad de Antofagasta, Avenida Angamos 601, Casilla 170, Antofagasta 1240000, Chile; e Department of Chemistry, M.M.A.M.C (Tribhuvan University) Biratnagar, Nepal; Katholieke Universiteit Leuven, Belgium

**Keywords:** crystal structure, 1*H*-pyrrole ring, hydrogen bonds, Hirshfeld surface analysis

## Abstract

In the title compound, C_15_H_14_N_2_O_2_·H_2_O, the 1*H*-pyrrole ring makes a dihedral angle of 59.95 (13)° with the phenyl ring. In the crystal, the mol­ecules are connected by C—H⋯O hydrogen bonds into layers parallel to the (020) plane, while two mol­ecules are connected to the water mol­ecule by two N—H⋯O hydrogen bonds and one mol­ecule by an O—H⋯O hydrogen bond. C—H⋯π and π–π inter­actions further link the mol­ecules into chains extending in the [  01] direction and stabilize the mol­ecular packing. According to a Hirshfeld surface study, H⋯H (49.4%), C⋯H/H⋯C (23.2%) and O⋯H/H⋯O (20.0%) inter­actions are the most significant contributors to the crystal packing.

## Abstract 2

In the title compound, the mol­ecules are connected by C—H⋯O hydrogen bonds in layers parallel to the (020) plane. C—H⋯π and π–π inter­actions further link the mol­ecules into chains extending in the [ 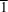 01] direction.

## 1. Chemical context

Heterocyclic and carbocyclic aromatic systems are the most important compounds in organic chemistry (Gurbanov *et al.*, 2017 ▸; Aliyeva *et al.*, 2023 ▸). Organic synthesis is developing enormously with newer aromatic compounds having been obtained for diverse medicinal and commercial purposes (Maharramov *et al.*, 2021 ▸; Poustforoosh *et al.*, 2022 ▸; Gurbanov *et al.*, 2022 **a* ▸,b* ▸). Nowadays, the application of five and six-membered heterocycles in particular has been expanded in different branches of chemistry, including coordination chemistry (Gurbanov *et al.*, 2021 ▸; Mahmoudi *et al.*, 2021 ▸), drug design and development (Çelik *et al.*, 2023 ▸) and material science (Velásquez *et al.*, 2019 ▸; Afkhami *et al.*, 2019 ▸). The pyrrole core is the most common five-membered heteroaromatic ring system in nitro­gen heterocycles (Mahmoudi *et al.*, 2017 ▸). It is an essential structural motif present in many natural tetra­pyrrole scaffolds of heme and related cofactors (chloro­phyll *a*, heme *b*, vitamin B_12_, factor 430) and other bioactive mol­ecules such as porphobilinogen, nargenicin and prodigiosin (Walsh *et al.*, 2006 ▸). The combination of different pharmacophores in a pyrrole ring system has led to the formation of more active compounds, such as elopiprazole, lorpiprazole, isamoltane, obatoclax (Bhardwaj *et al.*, 2015 ▸). On the other hand, there have been a variety of significant examples of pyrrole derivatives used as target products as well as synthetic inter­mediates (Naghiyev *et al.*, 2020 ▸, 2021 ▸, 2022 ▸). 
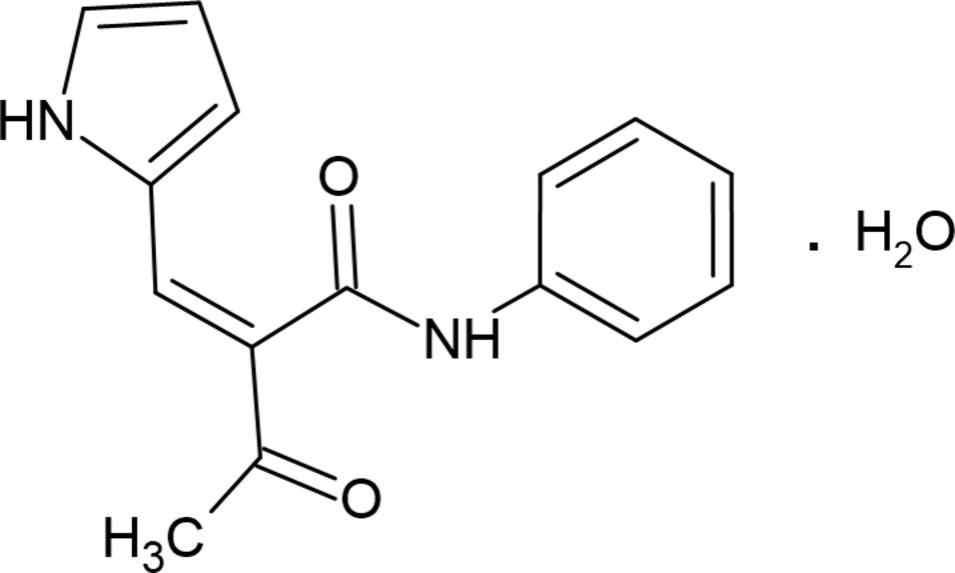


## 2. Structural commentary

**Figure 1:**
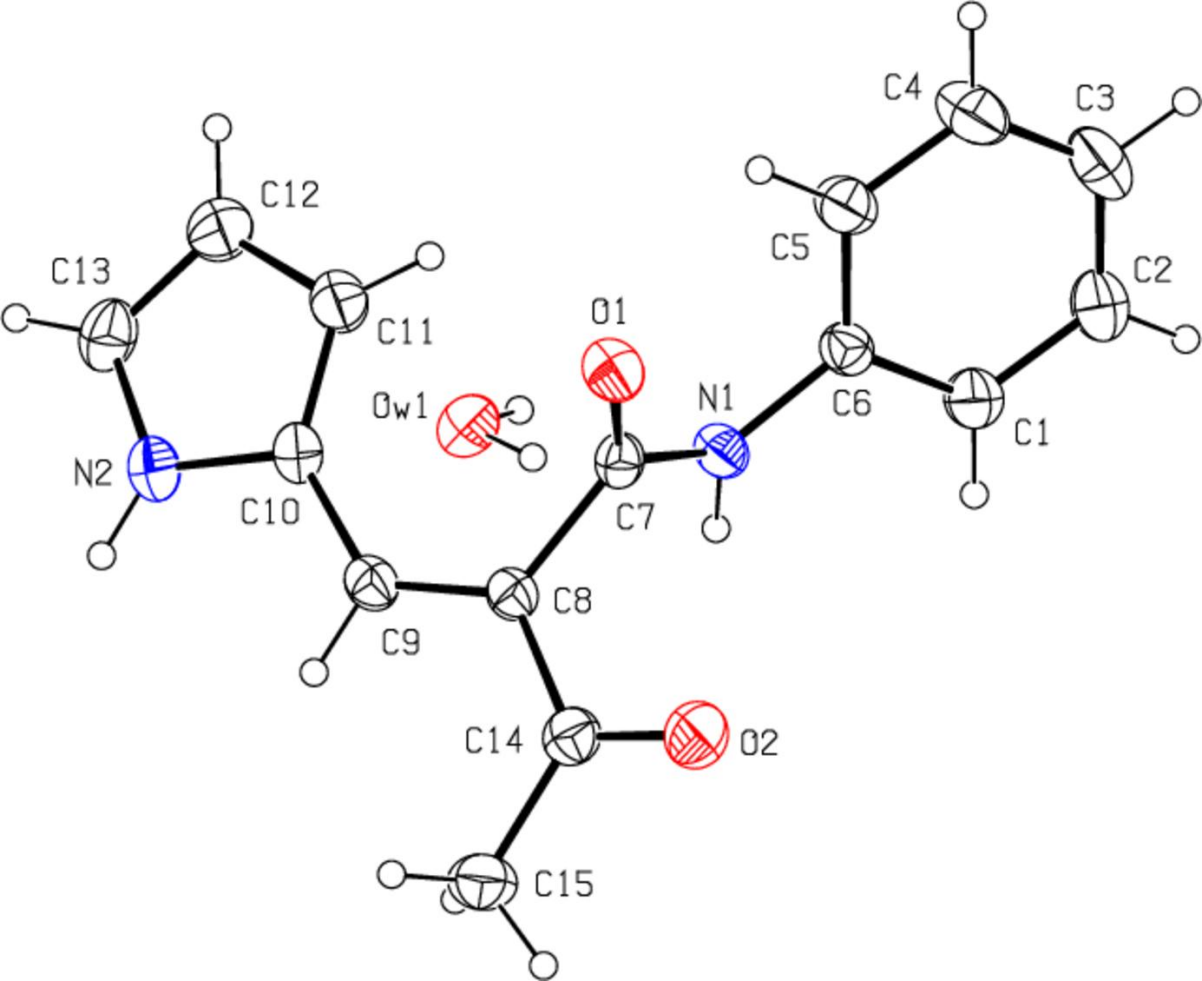
The mol­ecular structure of the title compound, showing the atom labelling and displacement ellipsoids drawn at the 30% probability level.

The title compound crystallizes with one water mol­ecule in the asymmetric unit (Fig. 1 ▸). The 1*H*-pyrrole ring (N2/C10–C13) makes a dihedral angle of 59.95 (13)° with the phenyl ring (C1–C6). The conformation is stabilized by an intra­molecular C5—H5⋯O1 inter­action (Table 1 ▸). In addition, an O*W*1—H*W*1⋯O1 hydrogen bond is observed between the main mol­ecule and the water mol­ecule in the asymmetric unit (Table 1 ▸). The 1*H*-pyrrole ring and *N*-phenyl­formamide substituents on the C8=C9 double bond are in a *cis* configuration [the C7—C8—C9—C10 torsion angle is 1.5 (3) °] and the 1*H*-pyrrole ring and the acetaldehyde substituents are in a *trans* configuration [the C14—C8—C9—C10 torsion angle is 179.17 (18) °].

The other torsion angles C5—C6—N1—C7, C6—N1—C7—O1, C6—N1—C7—C8, N1—C7—C8—C14, N1—C7—C8—C9, C7—C8—C14—C15 and C8—C9—C10—C11 are −30.7 (3), 6.7 (3), −172.19 (17), 85.2 (2), −97.0 (2), −176.03 (18) and −1.0 (4)°, respectively. The geometric parameters of the title compound are normal and comparable to that of related compound listed in the *Database survey* section.

## 3. Supra­molecular features and Hirshfeld surface analysis

**Figure 2:**
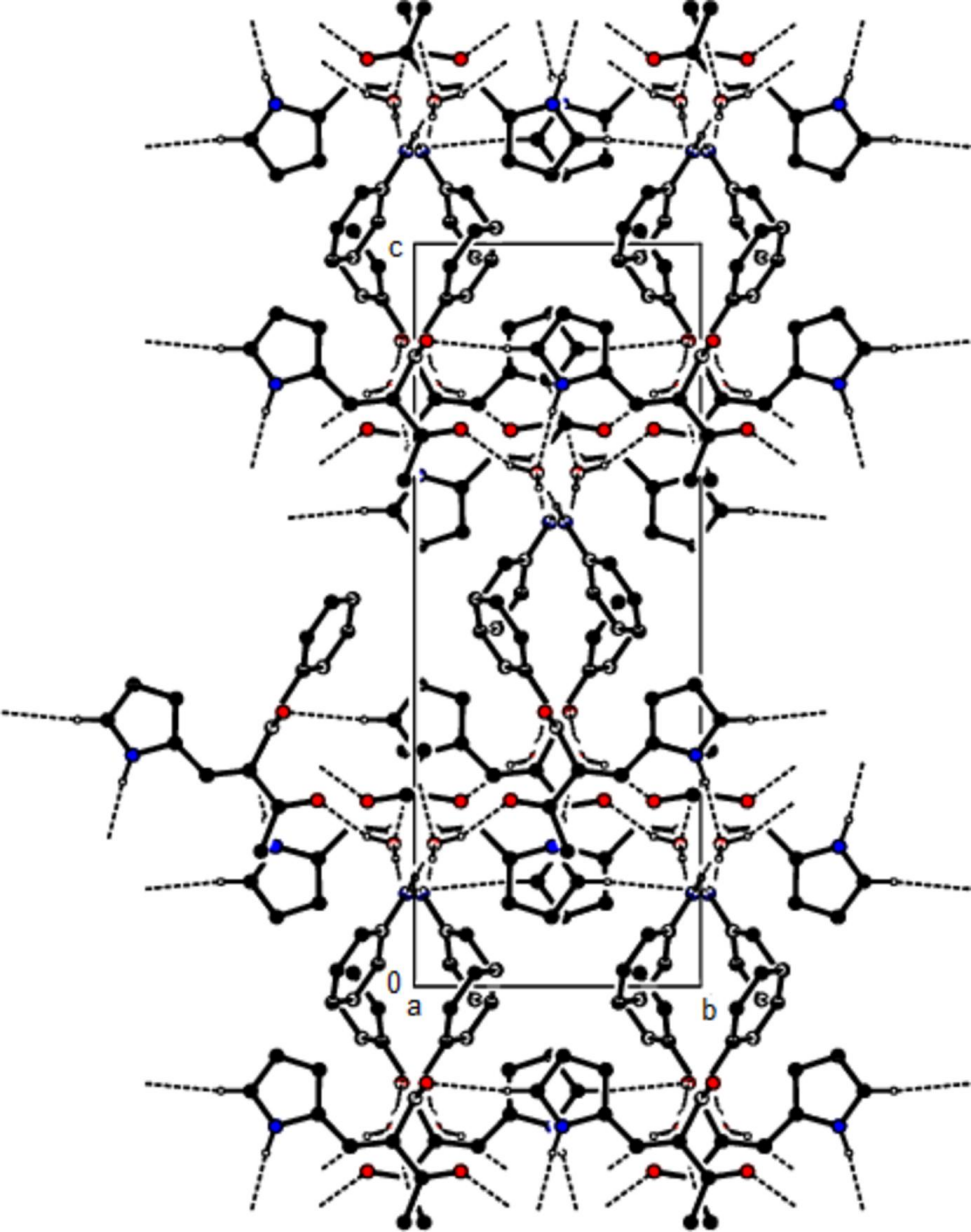
View of the crystal packing of the title compound along the *a*-axis showing N—H⋯O, C—H⋯O and O—H⋯O hydrogen bonds as dashed lines.

**Figure 4:**
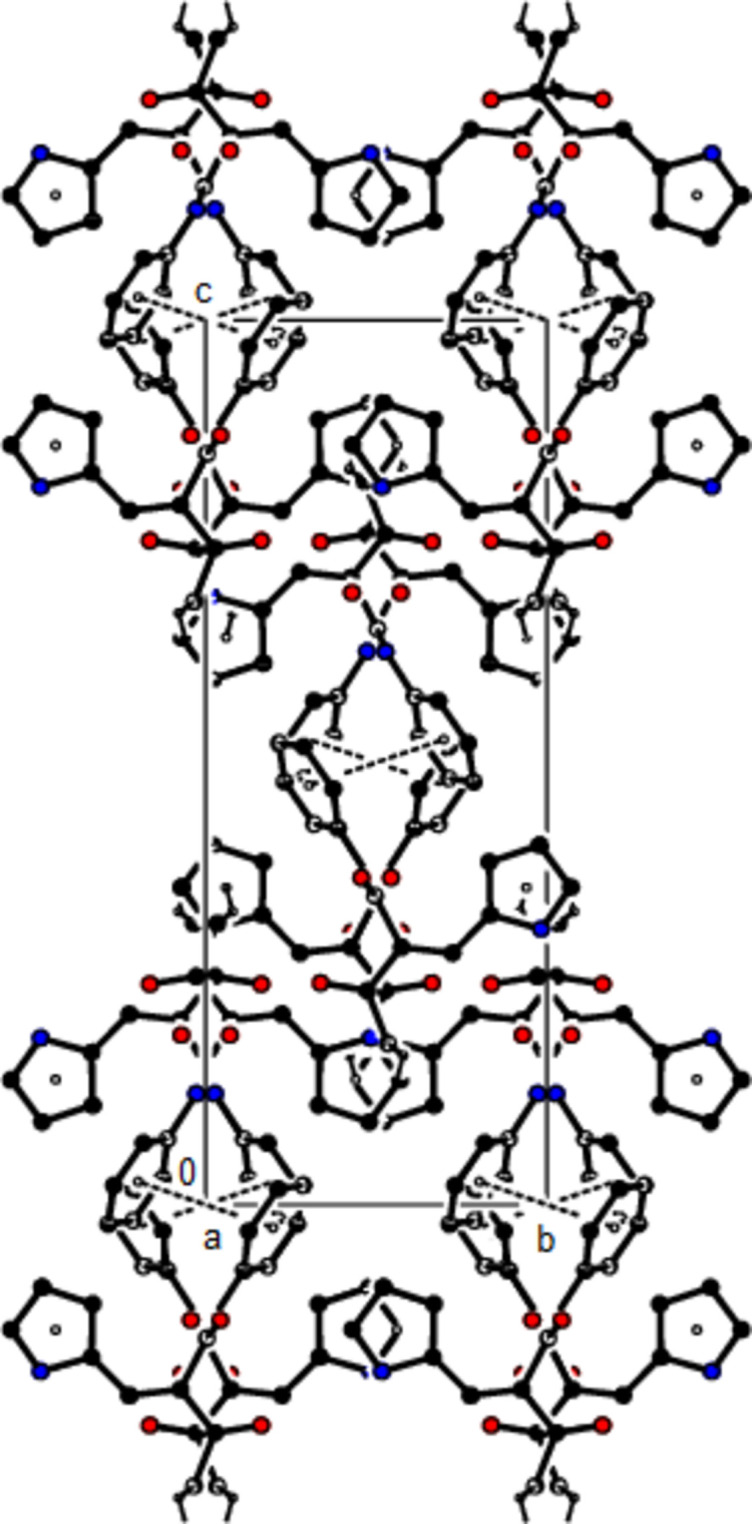
View of the crystal packing of the title compound along the *a*-axis showing the C—H⋯π and π–π inter­actions as dashed lines.

In the crystal, the mol­ecules are also connected by C—H⋯O hydrogen bonds in layers parallel to the (020) plane, while two mol­ecules are connected to the water mol­ecule by two N—H⋯O hydrogen bonds and one mol­ecule by an O—H⋯O hydrogen bond (Table 1 ▸, Figs. 2 ▸ and 3 ▸). C—H⋯π and π–π inter­actions [*Cg*2⋯*Cg*2(1 − *x*, 1 − *y*, 1 − *z*) = 3.8404 (16) Å, slippage = 0.858 Å; *Cg*2 is the centroid of phenyl ring C1–C6] link the mol­ecules into chains extending in the [ 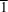 01] direction and stabilize the mol­ecular packing (Table 1 ▸, Figs. 4 ▸ and 5 ▸).

**Figure 6:**
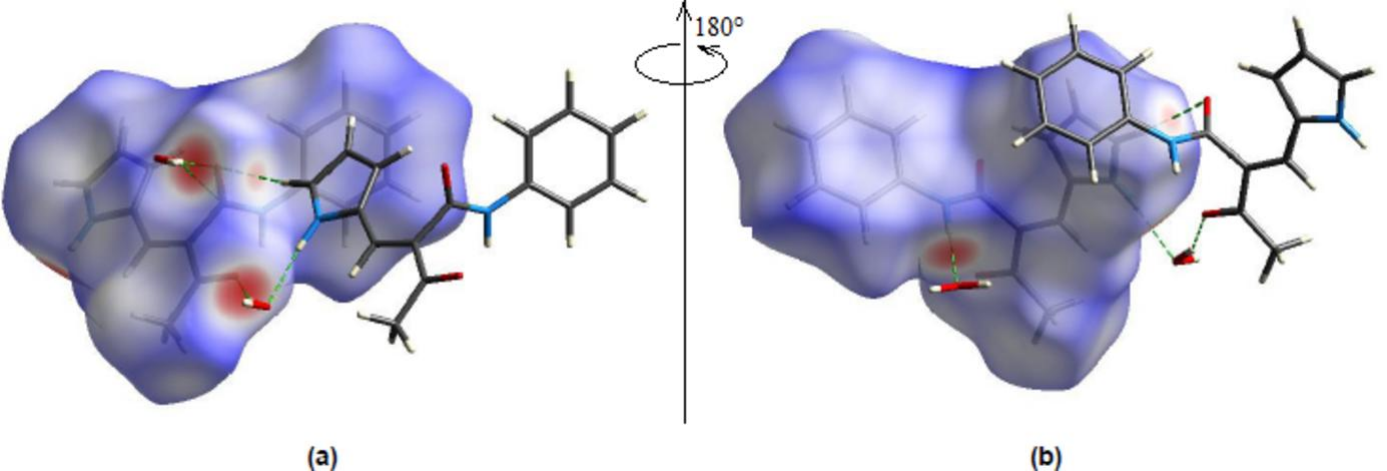
(*a*) Front and (*b*) back sides of the three-dimensional Hirshfeld surface of the title compound mapped over *d*_norm_, with a fixed colour scale of −0.6778 to +1.5015 a.u.

**Figure 7:**
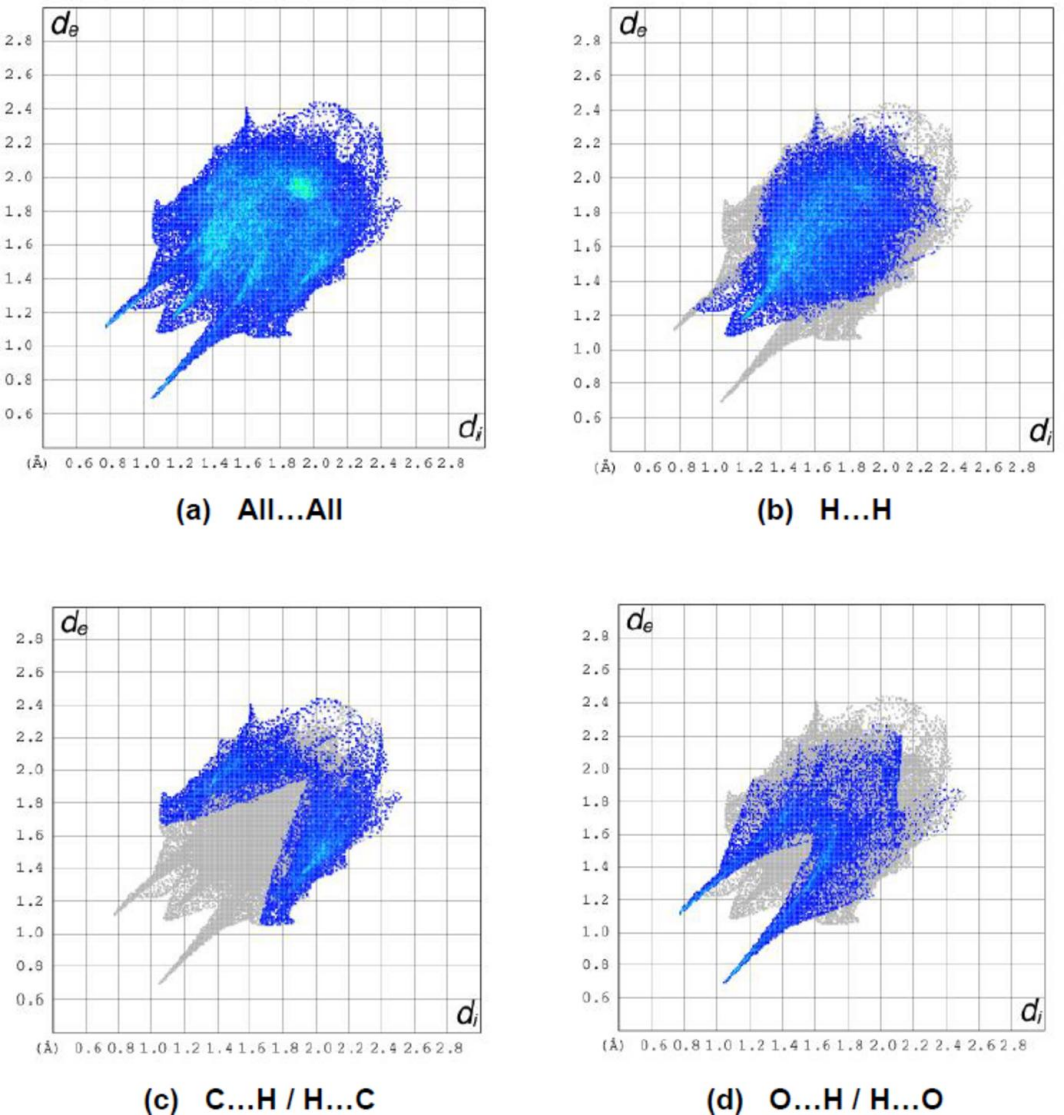
The two-dimensional fingerprint plots of the title compound, showing (*a*) all inter­actions, and delineated into (*b*) H⋯H, (*c*) C⋯H/H⋯C and (*d*) O⋯H/H⋯O inter­actions. [*d*_e_ and *d*_i_ represent the distances from a point on the Hirshfeld surface to the nearest atoms outside (external) and inside (inter­nal) the surface, respectively].

*Crystal Explorer 17.5* (Spackman *et al.*, 2021 ▸) was used to generate Hirshfeld surfaces and two-dimensional fingerprint plots in order to qu­antify the inter­molecular inter­actions in the crystal. The Hirshfeld surfaces were mapped over *d*_norm_ in the range −0.6778 (red) to +1.5015 (blue) a.u. (Fig. 6 ▸). The inter­actions given in Table 2 ▸ play a key role in the mol­ecular packing of the title compound. The most important inter­atomic contact is H⋯H as it makes the highest contribution to the crystal packing (49.4%, Fig. 7 ▸ *b*). Other major contributors are C⋯H/H⋯C (23.2%, Fig. 7 ▸ *c*) and O⋯H/H⋯O (20.0%, Fig. 7 ▸ *d*) inter­actions. Other smaller contributions are made by C⋯C (3.4%), N⋯H/H⋯N (3.3%), C⋯N/N⋯C (0.4%) and C⋯O/O⋯C (0.3%) inter­actions.

## 4. Database survey

A search of the Cambridge Structural Database (CSD, Version 5.43, last update November 2022; Groom *et al.*, 2016 ▸) for structures containing the fragment N—C—CH=C—C(=O)—NH, in which the N—C bond is part of a five-membered ring and the CH=C bond is acyclic, resulted in one hit, *N*-[(1,1-di­methyl­eth­oxy)carbon­yl]-l-alanyl-[(2*Z*)-3-(pyrrolidin-2-yl)-2-methyl-2-propeno­yl]-l-alanine methyl­amide di­chloro­methane solvate hydrate (CSD refcode SEFCUC; Grison *et al.*, 2005 ▸).

In the crystal of SEFCUC, mol­ecules are connected by N—H⋯O and C—H⋯O hydrogen bonds, forming mol­ecular layers parallel to the (001) plane. These layers are connected to each other by van der Waals forces. Torsion angles at the central C—C=C—C(=O)—NH unit in SEFCUC, *i.e*. the torsion angles C9—C13—C14—C16 and C13—C14—C16—N3 are −3.1 (5) and −53.1 (4)°, respectively. SEFCUC shows a folded conformation due to an intra­molecular N—H⋯O hydrogen bond. The amide group is *trans*-planar, as in the title compound.

## 5. Synthesis and crystallization

To a solution of pyrrole-2-carboxaldehyde (1 g, 10 mmol) and acetoacetanilide (1.77 g, 10 mmol) in ethanol (80%, 20 mL), were added methyl­piperazine (3–4 drops) and the mixture was stirred at room temperature for 2 h. The reaction mixture was then left overnight. The precipitated crystals were separated by filtration and recrystallized from an ethanol/water (1:1) solution (yield 69%; m.p. 513–514 K).

^1^H NMR (300 MHz, DMSO-*d*_6_, δ): 2.34 (*s*, 3H, CH_3_), 6.21 (*d*, 1H, CH_pyr._), 6.57 (1H, *d*, CH_pyr._), 7.10 (*t*, 1H, CH_pyr_), 7.14 (*t*, 1H, CH_arom._), 7.35 (*m*, 2H; 2CH_arom._), 7.57 (*s*, 1H, CH=), 7.70 (*d*, 2H, 2CH_arom._), 10.41 (*s*, 1H, NH), 11.52 (*s*, 1H, NH). ^13^C NMR (75 MHz, DMSO-*d*_6_ δ): 26.45 (CH_3_), 112.12 (CH_pyr._), 114.66 (CH_pyr._), 119.74 (2CH_arom._), 124.08 (CH_pyr._), 126.70 (CH_arom._), 129.37 (2CH_arom._), 130.66 (C_pyr._), 136.83 (CH=), 139.58 (C_quat._), 139.70 (C_quat._), 166.74 (C=O), 195.29 (C=O).

## 6. Refinement

Crystal data, data collection and structure refinement details are summarized in Table 3 ▸. The hydrogen atoms of the water mol­ecule and the hydrogen atoms bound to nitro­gen were located in difference-Fourier maps and refined with fixed positional thermal displacement parameters and with *U*_iso_(H) = 1.2*U*_eq_(N) or 1.5*U*_eq_(O). All carbon-bound hydrogen atoms were positioned geometrically (C—H = 0.93–0.96 Å) and were included in the refinement in the riding-model approximation with *U*_iso_(H) = 1.2 or 1.5*U*_eq_(C). One reflection (0 1 1), affected by the beam stop, was omitted in the final cycles of refinement. Owing to poor agreement between observed and calculated intensities, fourteen outliers ( 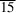 1 10, 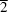 3 15, 8 4 16, 16 0 0, 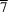 3 18, 0 5 23, 2 3 7, 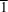 2 21, 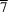 7 18, 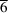 6 8, 1 5 18, 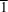 3 18, 0 2 14, 5 5 20) were omitted during the final refinement cycle. The value of *R*(int) should normally be considerably lower than 0.10. The value of *R*(int) of 0.205 in this study may be high due to poor crystal quality.

## Supplementary Material

Crystal structure: contains datablock(s) I. DOI: 10.1107/S2056989023009799/vm2291sup1.cif

Structure factors: contains datablock(s) I. DOI: 10.1107/S2056989023009799/vm2291Isup2.hkl

Supporting information file. DOI: 10.1107/S2056989023009799/vm2291Isup3.cml

CCDC reference: 2306713

Additional supporting information:  crystallographic information; 3D view; checkCIF report

## Figures and Tables

**Figure 3:**
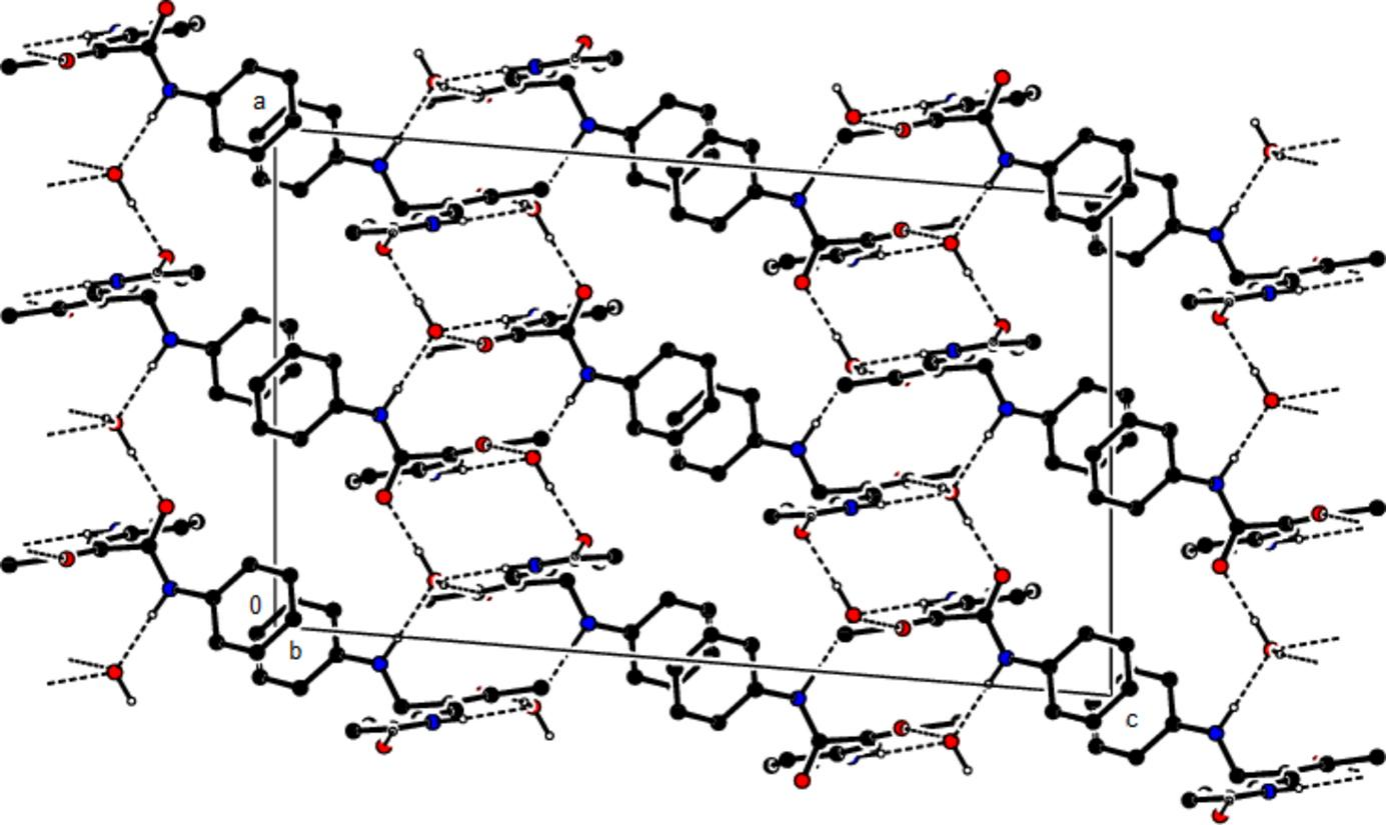
View of the crystal packing of the title compound along the *b*-axis showing N—H⋯O, C—H⋯O and O—H⋯O hydrogen bonds as dashed lines.

**Figure 5:**
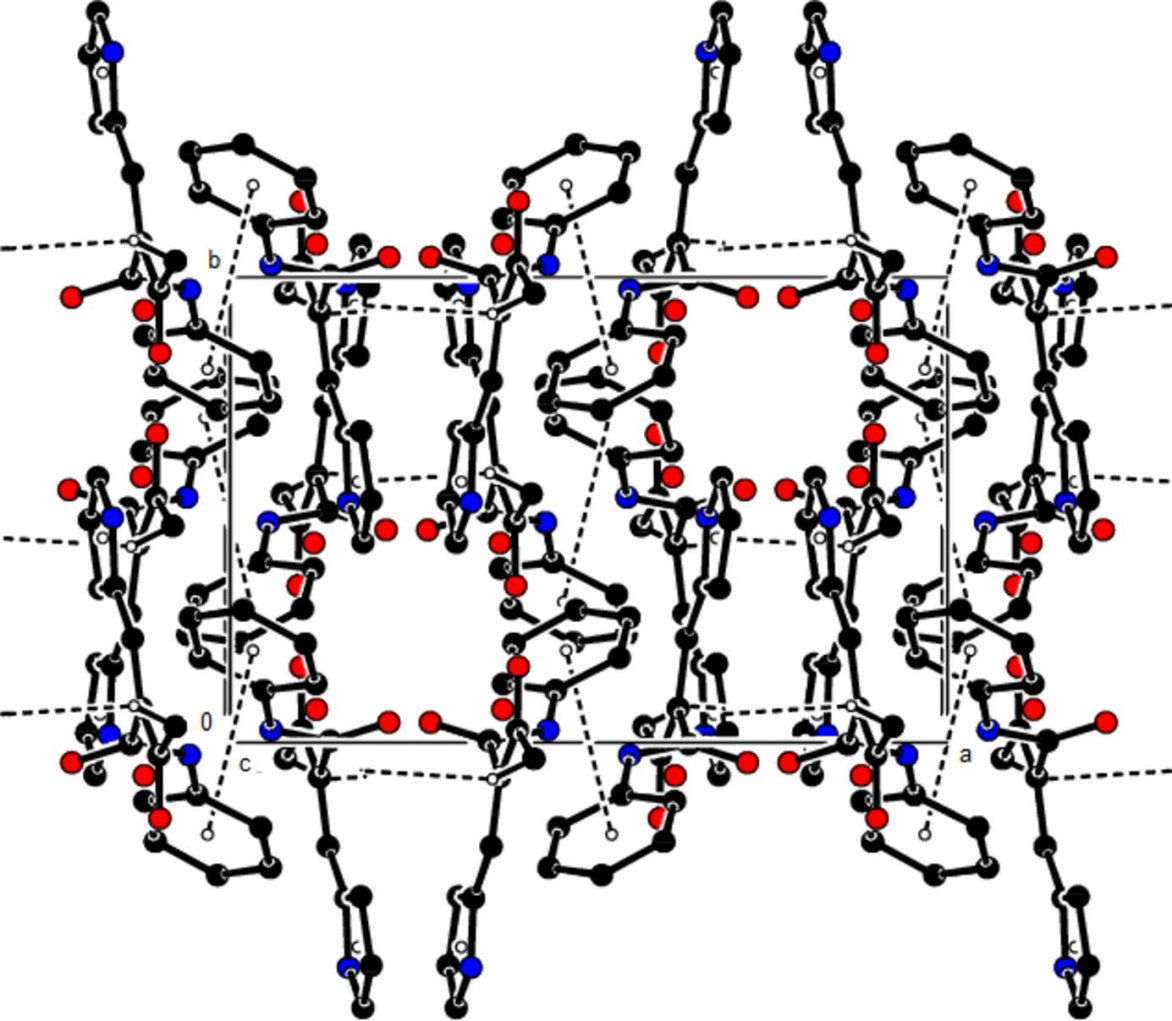
View of the crystal packing of the title compound along the *c*-axis showing the C—H⋯π and π–π inter­actions as dashed lines.

**Table 1:** Hydrogen-bond geometry (Å, °) *Cg*1 is the centroid of the N2/C10–C13 pyrrole ring.

*D*—H⋯*A*	*D*—H	H⋯*A*	*D*⋯*A*	*D*—H⋯*A*
C5—H5⋯O1	0.93	2.41	2.906 (3)	113
C13—H13⋯O1^i^	0.93	2.56	3.480 (3)	173
N1—H*N*1⋯O*W*1^ii^	0.91 (2)	1.99 (2)	2.898 (2)	179 (2)
N2—H*N*2⋯O*W*1^iii^	0.89 (2)	2.02 (2)	2.901 (2)	173 (2)
O*W*1—H*W*1⋯O1	0.92 (3)	1.80 (3)	2.718 (2)	177 (2)
O*W*1—H*W*2⋯O2^iv^	0.87 (3)	1.92 (3)	2.750 (2)	160 (2)
C15—H15*C*⋯*Cg*1^iii^	0.96	2.66	3.536 (3)	151

Symmetry codes: (i) 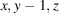 ; (ii) 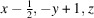 ; (iii) 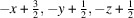 ; (iv) 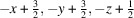 .

**Table 2:** Summary of short inter­atomic contacts (Å) in the title compound

Contact	Distance	Symmetry operation
O1⋯H*W*1	1.80	*x*, *y*, *z*
O1⋯H13	2.56	*x*, 1 + *y*, *z*
O2⋯H*W*2	1.92	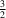 − *x*, 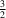 − *y*, 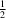 − *z*
H*N*1⋯O*W*1	1.99	− 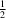 + *x*, 1 − *y*, *z*
N2⋯H15*B*	2.92	1 − *x*, − 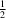 + *y*, 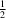 − *z*
H*N*2⋯O*W*1	2.02	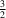 − *x*, 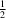 − *y*, 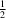 − *z*
H15*C*⋯N2	2.76	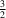 − *x*, 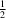 − *y*, 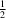 − *z*
H5⋯H5	2.36	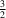 − *x*, *y*, 1 − *z*
H3⋯C11	3.06	1 − *x*, 1 − *y*, 1 − *z*
H3⋯H15*A*	2.59	*x*, 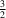 − *y*, 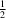 + *z*

**Table 3:** Experimental details

Crystal data	
Chemical formula	C_15_H_14_N_2_O_2_·H_2_O
*M*_r_	272.30
Crystal system, space group	Monoclinic, *I*2/*a*
Temperature (K)	294
*a*, *b*, *c* (Å)	13.7420 (13), 8.8912 (13), 23.114 (2)
β (°)	94.742 (4)
*V* (Å^3^)	2814.5 (6)
*Z*	8
Radiation type	Mo *K*α
μ (mm^−1^)	0.09
Crystal size (mm)	0.29 × 0.24 × 0.21

Data collection	
Diffractometer	Bruker APEXII CCD
No. of measured, independent and observed [*I* > 2σ(*I*)] reflections	98978, 2677, 1729
*R*_int_	0.205
(sin θ/λ)_max_ (Å^−1^)	0.611

Refinement	
*R*[*F* ^2^ > 2σ(*F* ^2^)], *wR*(*F* ^2^), *S*	0.041, 0.109, 1.05
No. of reflections	2677
No. of parameters	194
H-atom treatment	H atoms treated by a mixture of independent and constrained refinement
Δρ_max_, Δρ_min_ (e Å^−3^)	0.14, −0.18

Computer programs: *APEX2* and *SAINT* (Bruker, 2018 ▸), *SHELXT2014/5* (Sheldrick, 2015*a* ▸), *SHELXL2018/3* (Sheldrick, 2015*b* ▸), *ORTEP-3 for Windows* (Farrugia, 2012 ▸) and *PLATON* (Spek, 2020 ▸).

## supplementary crystallographic information

### Crystal data

**Table:**

C_15_H_14_N_2_O_2_·H_2_O	*F*(000) = 1152
*M**_r_* = 272.30	*D*_x_ = 1.285 Mg m^−^^3^
Monoclinic, *I*2/*a*	Mo *K*α radiation, λ = 0.71073 Å
*a* = 13.7420 (13) Å	Cell parameters from 7717 reflections
*b* = 8.8912 (13) Å	θ = 3.2–21.8°
*c* = 23.114 (2) Å	µ = 0.09 mm^−^^1^
β = 94.742 (4)°	*T* = 294 K
*V* = 2814.5 (6) Å^3^	Prism, colourless
*Z* = 8	0.29 × 0.24 × 0.21 mm

### Data collection

**Table:**

Bruker APEXII CCD diffractometer	*R*_int_ = 0.205
φ and ω scans	θ_max_ = 25.7°, θ_min_ = 2.7°
98978 measured reflections	*h* = −16→16
2677 independent reflections	*k* = −10→10
1729 reflections with *I* > 2σ(*I*)	*l* = −28→28

### Refinement

**Table:**

Refinement on *F*^2^	0 restraints
Least-squares matrix: full	Hydrogen site location: mixed
*R*[*F*^2^ > 2σ(*F*^2^)] = 0.041	H atoms treated by a mixture of independent and constrained refinement
*wR*(*F*^2^) = 0.109	*w* = 1/[σ^2^(*F*_o_^2^) + (0.0357*P*)^2^ + 2.2064*P*] where *P* = (*F*_o_^2^ + 2*F*_c_^2^)/3
*S* = 1.05	(Δ/σ)_max_ < 0.001
2677 reflections	Δρ_max_ = 0.14 e Å^−^^3^
194 parameters	Δρ_min_ = −0.18 e Å^−^^3^

### Special details

**Table:**

Geometry. All esds (except the esd in the dihedral angle between two l.s. planes) are estimated using the full covariance matrix. The cell esds are taken into account individually in the estimation of esds in distances, angles and torsion angles; correlations between esds in cell parameters are only used when they are defined by crystal symmetry. An approximate (isotropic) treatment of cell esds is used for estimating esds involving l.s. planes.

### Fractional atomic coordinates and isotropic or equivalent isotropic displacement parameters (Å^2^)

**Table:**

	*x*	*y*	*z*	*U*_iso_*/*U*_eq_	
C1	0.45829 (16)	0.6822 (3)	0.43032 (9)	0.0540 (6)	
H1	0.408073	0.670947	0.401110	0.065*	
C2	0.4445 (2)	0.7687 (3)	0.47832 (10)	0.0710 (7)	
H2	0.384992	0.816610	0.481260	0.085*	
C3	0.5178 (2)	0.7850 (3)	0.52188 (11)	0.0699 (7)	
H3	0.508515	0.845175	0.553880	0.084*	
C4	0.60437 (19)	0.7124 (3)	0.51790 (10)	0.0669 (7)	
H4	0.653634	0.721325	0.547795	0.080*	
C5	0.61958 (16)	0.6259 (3)	0.47001 (9)	0.0532 (6)	
H5	0.678910	0.576957	0.467656	0.064*	
C6	0.54696 (14)	0.6122 (2)	0.42572 (8)	0.0388 (5)	
C7	0.64024 (14)	0.5036 (2)	0.34901 (8)	0.0372 (4)	
C8	0.62601 (13)	0.4259 (2)	0.29110 (8)	0.0361 (4)	
C9	0.63778 (13)	0.2768 (2)	0.28452 (8)	0.0398 (5)	
H9	0.628673	0.241390	0.246603	0.048*	
C10	0.66233 (14)	0.1657 (2)	0.32780 (8)	0.0415 (5)	
C11	0.68283 (17)	0.1689 (3)	0.38755 (9)	0.0547 (6)	
H11	0.686480	0.254312	0.410856	0.066*	
C12	0.69700 (18)	0.0209 (3)	0.40637 (10)	0.0614 (6)	
H12	0.711383	−0.010172	0.444544	0.074*	
C13	0.68602 (17)	−0.0701 (3)	0.35893 (10)	0.0571 (6)	
H13	0.691324	−0.174348	0.359127	0.069*	
C14	0.60180 (14)	0.5269 (2)	0.24196 (8)	0.0445 (5)	
C15	0.57908 (17)	0.4655 (3)	0.18195 (9)	0.0588 (6)	
H15A	0.556811	0.545433	0.156270	0.088*	
H15B	0.529029	0.390312	0.182627	0.088*	
H15C	0.636866	0.421282	0.168541	0.088*	
N1	0.55800 (12)	0.52714 (18)	0.37468 (7)	0.0397 (4)	
HN1	0.5032 (16)	0.497 (2)	0.3534 (9)	0.048*	
N2	0.66617 (13)	0.01667 (19)	0.31163 (8)	0.0472 (4)	
HN2	0.6547 (16)	−0.017 (2)	0.2755 (10)	0.057*	
O1	0.72199 (10)	0.54315 (16)	0.36963 (6)	0.0486 (4)	
O2	0.60026 (13)	0.66292 (18)	0.25062 (6)	0.0653 (5)	
OW1	0.88137 (11)	0.57116 (18)	0.30826 (6)	0.0496 (4)	
HW1	0.8260 (19)	0.564 (3)	0.3282 (10)	0.074*	
HW2	0.8891 (18)	0.664 (3)	0.2981 (11)	0.074*	

### Atomic displacement parameters (Å^2^)

**Table:**

	*U*^11^	*U*^22^	*U*^33^	*U*^12^	*U*^13^	*U*^23^
C1	0.0510 (13)	0.0643 (14)	0.0468 (12)	0.0125 (11)	0.0048 (10)	−0.0042 (11)
C2	0.0739 (17)	0.0797 (18)	0.0607 (16)	0.0279 (14)	0.0127 (13)	−0.0136 (14)
C3	0.0875 (19)	0.0703 (17)	0.0534 (14)	0.0020 (14)	0.0149 (13)	−0.0227 (13)
C4	0.0690 (16)	0.0836 (19)	0.0474 (13)	−0.0054 (14)	0.0004 (11)	−0.0176 (13)
C5	0.0487 (12)	0.0657 (15)	0.0445 (12)	0.0039 (11)	0.0004 (10)	−0.0096 (11)
C6	0.0448 (11)	0.0374 (11)	0.0347 (10)	−0.0020 (9)	0.0056 (8)	−0.0001 (8)
C7	0.0404 (11)	0.0299 (10)	0.0414 (11)	0.0011 (8)	0.0053 (8)	0.0011 (8)
C8	0.0360 (10)	0.0380 (11)	0.0348 (10)	−0.0003 (8)	0.0054 (8)	−0.0022 (8)
C9	0.0381 (10)	0.0442 (12)	0.0376 (11)	−0.0006 (9)	0.0058 (8)	−0.0054 (9)
C10	0.0440 (11)	0.0355 (11)	0.0460 (12)	0.0030 (9)	0.0086 (9)	−0.0039 (9)
C11	0.0725 (15)	0.0457 (13)	0.0462 (13)	0.0066 (11)	0.0074 (11)	−0.0015 (10)
C12	0.0794 (17)	0.0542 (15)	0.0508 (13)	0.0089 (12)	0.0066 (12)	0.0085 (12)
C13	0.0652 (15)	0.0375 (12)	0.0696 (16)	0.0041 (11)	0.0108 (12)	0.0073 (12)
C14	0.0416 (11)	0.0487 (14)	0.0439 (12)	0.0040 (9)	0.0071 (9)	0.0034 (10)
C15	0.0593 (14)	0.0750 (16)	0.0414 (12)	0.0077 (12)	0.0007 (10)	0.0015 (11)
N1	0.0361 (9)	0.0454 (10)	0.0376 (9)	−0.0012 (7)	0.0030 (7)	−0.0074 (8)
N2	0.0526 (10)	0.0371 (10)	0.0526 (11)	0.0007 (8)	0.0076 (8)	−0.0068 (9)
O1	0.0381 (8)	0.0556 (9)	0.0523 (8)	−0.0057 (7)	0.0049 (6)	−0.0094 (7)
O2	0.0966 (13)	0.0433 (10)	0.0563 (10)	0.0071 (8)	0.0070 (8)	0.0089 (8)
OW1	0.0450 (8)	0.0511 (9)	0.0533 (9)	0.0040 (7)	0.0078 (7)	0.0091 (7)

### Geometric parameters (Å, º)

**Table:**

C1—C2	1.376 (3)	C9—H9	0.9300
C1—C6	1.380 (3)	C10—N2	1.379 (3)
C1—H1	0.9300	C10—C11	1.387 (3)
C2—C3	1.371 (4)	C11—C12	1.395 (3)
C2—H2	0.9300	C11—H11	0.9300
C3—C4	1.363 (4)	C12—C13	1.361 (3)
C3—H3	0.9300	C12—H12	0.9300
C4—C5	1.378 (3)	C13—N2	1.347 (3)
C4—H4	0.9300	C13—H13	0.9300
C5—C6	1.375 (3)	C14—O2	1.226 (2)
C5—H5	0.9300	C14—C15	1.500 (3)
C6—N1	1.420 (2)	C15—H15A	0.9600
C7—O1	1.235 (2)	C15—H15B	0.9600
C7—N1	1.336 (2)	C15—H15C	0.9600
C7—C8	1.505 (3)	N1—HN1	0.91 (2)
C8—C9	1.346 (3)	N2—HN2	0.89 (2)
C8—C14	1.465 (3)	OW1—HW1	0.92 (3)
C9—C10	1.427 (3)	OW1—HW2	0.87 (3)
			
C2—C1—C6	119.7 (2)	N2—C10—C9	119.18 (18)
C2—C1—H1	120.2	C11—C10—C9	134.50 (19)
C6—C1—H1	120.2	C10—C11—C12	107.6 (2)
C3—C2—C1	120.7 (2)	C10—C11—H11	126.2
C3—C2—H2	119.7	C12—C11—H11	126.2
C1—C2—H2	119.7	C13—C12—C11	107.8 (2)
C4—C3—C2	119.5 (2)	C13—C12—H12	126.1
C4—C3—H3	120.3	C11—C12—H12	126.1
C2—C3—H3	120.3	N2—C13—C12	108.4 (2)
C3—C4—C5	120.6 (2)	N2—C13—H13	125.8
C3—C4—H4	119.7	C12—C13—H13	125.8
C5—C4—H4	119.7	O2—C14—C8	118.96 (18)
C6—C5—C4	119.9 (2)	O2—C14—C15	120.36 (19)
C6—C5—H5	120.0	C8—C14—C15	120.68 (19)
C4—C5—H5	120.0	C14—C15—H15A	109.5
C5—C6—C1	119.57 (18)	C14—C15—H15B	109.5
C5—C6—N1	123.05 (18)	H15A—C15—H15B	109.5
C1—C6—N1	117.37 (17)	C14—C15—H15C	109.5
O1—C7—N1	124.06 (18)	H15A—C15—H15C	109.5
O1—C7—C8	121.43 (16)	H15B—C15—H15C	109.5
N1—C7—C8	114.50 (16)	C7—N1—C6	127.24 (17)
C9—C8—C14	122.57 (18)	C7—N1—HN1	114.1 (13)
C9—C8—C7	123.00 (17)	C6—N1—HN1	117.9 (13)
C14—C8—C7	114.39 (16)	C13—N2—C10	109.86 (18)
C8—C9—C10	128.81 (18)	C13—N2—HN2	125.1 (15)
C8—C9—H9	115.6	C10—N2—HN2	125.0 (15)
C10—C9—H9	115.6	HW1—OW1—HW2	109 (2)
N2—C10—C11	106.30 (18)		
			
C6—C1—C2—C3	−0.6 (4)	N2—C10—C11—C12	−1.0 (2)
C1—C2—C3—C4	−1.2 (4)	C9—C10—C11—C12	177.1 (2)
C2—C3—C4—C5	1.5 (4)	C10—C11—C12—C13	0.4 (3)
C3—C4—C5—C6	−0.1 (4)	C11—C12—C13—N2	0.3 (3)
C4—C5—C6—C1	−1.6 (3)	C9—C8—C14—O2	−173.92 (19)
C4—C5—C6—N1	178.9 (2)	C7—C8—C14—O2	3.9 (3)
C2—C1—C6—C5	2.0 (3)	C9—C8—C14—C15	6.2 (3)
C2—C1—C6—N1	−178.5 (2)	C7—C8—C14—C15	−176.03 (18)
O1—C7—C8—C9	84.1 (2)	O1—C7—N1—C6	6.7 (3)
N1—C7—C8—C9	−97.0 (2)	C8—C7—N1—C6	−172.19 (17)
O1—C7—C8—C14	−93.7 (2)	C5—C6—N1—C7	−30.7 (3)
N1—C7—C8—C14	85.2 (2)	C1—C6—N1—C7	149.9 (2)
C14—C8—C9—C10	179.17 (18)	C12—C13—N2—C10	−1.0 (3)
C7—C8—C9—C10	1.5 (3)	C11—C10—N2—C13	1.2 (2)
C8—C9—C10—N2	176.92 (19)	C9—C10—N2—C13	−177.18 (17)
C8—C9—C10—C11	−1.0 (4)		

### Hydrogen-bond geometry (Å, º)

*Cg*1 is the centroid of the N2/C10–C13 pyrrole ring.

**Table:**

*D*—H···*A*	*D*—H	H···*A*	*D*···*A*	*D*—H···*A*
C5—H5···O1	0.93	2.41	2.906 (3)	113
C13—H13···O1^i^	0.93	2.56	3.480 (3)	173
N1—H*N*1···O*W*1^ii^	0.91 (2)	1.99 (2)	2.898 (2)	179 (2)
N2—H*N*2···O*W*1^iii^	0.89 (2)	2.02 (2)	2.901 (2)	173 (2)
O*W*1—H*W*1···O1	0.92 (3)	1.80 (3)	2.718 (2)	177 (2)
O*W*1—H*W*2···O2^iv^	0.87 (3)	1.92 (3)	2.750 (2)	160 (2)
C15—H15*C*···*Cg*1^iii^	0.96	2.66	3.536 (3)	151

Symmetry codes: (i) *x*, *y*−1, *z*; (ii) *x*−1/2, −*y*+1, *z*; (iii) −*x*+3/2, −*y*+1/2, −*z*+1/2; (iv) −*x*+3/2, −*y*+3/2, −*z*+1/2.
